# Treatment and Outcomes among Patients with *Cryptococcus gattii* Infections in the United States Pacific Northwest

**DOI:** 10.1371/journal.pone.0088875

**Published:** 2014-02-19

**Authors:** Rachel M. Smith, Adamma Mba-Jonas, Mathieu Tourdjman, Trisha Schimek, Emilio DeBess, Nicola Marsden-Haug, Julie R. Harris

**Affiliations:** 1 Division of Foodborne, Waterborne and Environmental Diseases, Centers for Disease Control and Prevention, Atlanta, Georgia, United States of America; 2 Scientific Education and Professional Development Office, Centers for Disease Control and Prevention, Atlanta, Georgia, United States of America; 3 Oregon Public Health Division, Portland, Oregon, United States of America; 4 Washington State Department of Health, Shoreline, Washington, United States of America; University of Minnesota, United States of America

## Abstract

**Background:**

*Cryptococcus gattii* is a fungal pathogen causing an emerging outbreak in the United States Pacific Northwest (PNW). Treatment guidelines for cryptococcosis are primarily based on data from *C. neoformans* infections; applicability to PNW *C. gattii* infection is unknown. We evaluated the relationship between initial antifungal treatment and outcomes for PNW *C.gattii* patients.

**Methods:**

Cases were defined as culture-confirmed invasive *C. gattii* infections among residents of Oregon and Washington States during 2004–2011. Clinical data were abstracted from medical records through one year of follow-up. Recommended initial treatment for central nervous system (CNS), bloodstream, and severe pulmonary infections is amphotericin B and 5-flucytosine; for non-severe pulmonary infections, recommended initial treatment is fluconazole. Alternative initial treatment was defined as any other initial antifungal treatment.

**Results:**

Seventy patients survived to diagnosis; 50 (71%) received the recommended initial treatment and 20 (29%) received an alternative. Fewer patients with pulmonary infections [21 (64%)] than CNS infections [25 (83%)] received the recommended initial treatment (p = 0.07). Among patients with pulmonary infections, those with severe infections received the recommended initial treatment less often than those with non-severe infections (11% vs. 83%, p<0.0001). Eight patients with severe pulmonary infections received alternative initial treatments; three died. Four patients with non-severe pulmonary infections received alternative initial treatments; two died. There was a trend towards increased three-month mortality among patients receiving alternative vs. recommended initial treatment (30% vs. 14%, p = 0.12), driven primarily by increased mortality among patients with pulmonary disease receiving alternative vs. recommended initial treatment (42% vs. 10%, p = 0.07).

**Conclusions:**

*C.gattii* patients with pulmonary infections – especially severe infections – may be less likely to receive recommended treatment than those with CNS infections; alternative treatment may be associated with increased mortality. Reasons for receipt of alternative treatment among *C.gattii* patients in this area should be investigated, and clinician awareness of recommended treatment reinforced.

## Introduction

*Cryptococcus spp.* are basidiomycetous yeast, with two species, *C. gattii* and *C. neoformans*, causing nearly all human cryptococcal infections [1]–[3]. *C. neoformans* typically causes disease in immunocompromised individuals [4], [5], and is an important and common cause of opportunistic infections in HIV/AIDS patients worldwide [6]–[8]. In contrast, *C. gattii* has historically been characterized as a rare pathogen, with disease confined to tropical and subtropical climates, particularly the highly endemic regions of Australia and Papua New Guinea [9], [10]. Until recently, *C. gattii* was thought to affect primarily immunocompetent persons living in these regions [11]–[15].

Since 2004, an outbreak of *C. gattii* infections has been documented in the United States Pacific Northwest states of Oregon and Washington [16]. The outbreak in these states is thought to have originated in, and spread from, British Columbia, Canada, where infections caused by the same *C. gattii* genetic types have been documented since 1999 [17], [18]. Genetic sequencing has demonstrated that *C. gattii* can be divided into four molecular types, denoted as VGI, VGII, VGIII and VGIV [19]; these molecular types can be distinguished by various genetic techniques and have different geographic distributions [20]–[23]. The emergence of *C. gattii* infections in Oregon, Washington State, and British Columbia is primarily due to the clonal expansion of three genetic subtypes belonging to the molecular type VGII, called VGIIa, VGIIb, and VGIIc [24]–[26]; these have been referred to as ‘outbreak-strain’ subtypes. Outbreak-strain subtypes are different from those found in historically endemic Australia and Papua New Guinea (and other areas of the world), where infections are most frequently caused by nonclonal strains of *C. gattii*, most commonly VGI [11], [27]–[29]. Clinical differences between *C. gattii* infections in the United States Pacific Northwest and historically endemic areas have been described. While *C. gattii* in historically endemic areas has been reported to infect primarily immunocompetent persons, causing meningoencephalitis [11], [27], [28], [30]–[32], *C. gattii* infections in Oregon and Washington State occur frequently in immunocompromised persons and present most often as respiratory illness [33].

Current guidelines for the management and treatment of cryptococcal disease from the Infectious Disease Society of America (IDSA) recommend antifungal treatment that varies by site and severity of infection [34]. The recommended initial treatment (RIT) for severe pulmonary disease, central nervous system (CNS) disease, and other disseminated disease (such as cryptococcemia) is amphotericin B (AMB) and 5-flucytosine (5FC); for non-severe pulmonary disease, the RIT is fluconazole, with itraconazole and posaconazole as acceptable second-line agents [34]. These recommendations are unchanged from previous IDSA guidelines for cryptococcosis, released in 2000, which were available when the majority of patients in this report were diagnosed [35].

Although IDSA guidelines for treatment of cryptococcal disease are based primarily on data from *C. neoformans* infections in HIV and solid organ transplant patients, these guidelines are intended to apply to patients with *C. neoformans* or *C. gattii* infections. A limited number of *C. gattii*-specific recommendations were included for the first time in the 2010 IDSA guidelines and are based on data from *C. gattii* infections in historically endemic areas, the only data available at the time of the guideline-writing [34]. These recommendations pertain mainly to patients with cryptococcomas, which previous data have suggested are more common in patients infected with *C. gattii* than *C. neoformans* [31], and include consideration of surgery for patients with large cryptococcomas, increased radiologic and follow-up evaluations for those with cryptococcomas or hydrocephalus, and possible use of AMB/5FC in patients with large and/or multiple pulmonary cryptococcomas [34].

Given the genotypic and clinical differences between *C. gattii* infections in the United States Pacific Northwest and cryptococcal infections due to either *C. gattii* from historically endemic areas or *C. neoformans*, applicability of the current IDSA guidelines to *C. gattii* patients in Oregon and Washington State is unknown. We conducted a retrospective cohort study of *C. gattii* infections reported in these states to evaluate the relationship between IDSA guideline-recommended initial antifungal treatment and clinical outcomes.

## Methods

### Case Definition and Case-finding

A case was defined as culture-confirmed *C. gattii* infection reported to CDC during January 1, 2004 to October 1, 2011, in a person residing in Oregon or Washington State. Patients were identified from existing passive laboratory-based surveillance systems that capture culture-confirmed *C. gattii* infections in these states. Although reporting is passive, *C. gattii* has been notifiable in Oregon since 2011, and in Washington State (as a rare disease of public health importance) since 2006. This investigation was conducted as part of routine public health practice in response to an ongoing outbreak of *C. gattii* in the United States Pacific Northwest. This investigation was reviewed and designated as non-research by a CDC ethics liaison, informed consent was not obtained from patients, and the study was deemed exempt from formal institutional review board evaluation.

### Data Collection

We abstracted data from case-patient medical records using a standardized form. Information about demographics, underlying medical conditions, current medications, signs and symptoms, laboratory and radiologic studies, and treatments and procedures performed secondary to *C. gattii* diagnosis were recorded. Data were collected from case-patients’ initial visits and at two, six, 12, 24 and 52 weeks of follow-up. Death databases were searched to identify any deaths that occurred during follow-up. All data were entered into a Microsoft Access database.

### Definition of Terms

We limited our analyses to case-patients with invasive *C. gattii* disease, which we defined as infection of any of the deep organs or body tissues, including blood. We excluded superficial infections, including skin, throat and urinary tract infections without evidence of involvement of other organs, due to the small number and absence of specific treatment recommendations for these infections [34]. Additionally, we excluded children <15 years, as the IDSA guidelines for children differ to some extent from those for adults [34], [35].

Infections were categorized in a hierarchy, based on site of infection. Infections were categorized as ‘bloodstream’ if the patient had at least one positive blood culture for *C. gattii*, regardless of other positive cultures. Infections were categorized as ‘CNS’ if cerebrospinal fluid (CSF) or brain tissue cultures yielded *C. gattii* in the absence of documented bloodstream infection. Infections were also categorized as ‘CNS’ if the case-patient had a positive CSF cryptococcal antigen result or had brain tissue histopathology consistent with cryptococcal disease *and* a positive culture for *C. gattii* from a body site outside the CNS (not including blood). Infections were categorized as ‘pulmonary’ if respiratory specimens (sputum, bronchoalveolar lavage) or lung tissue cultures yielded *C. gattii* in the absence of documented bloodstream or CNS infection. Bloodstream infections were categorized separately from other invasive *C. gattii* infections (e.g. CNS infections) due to the high mortality that was observed in patients with fungemia due to *C. gattii.* Pulmonary infections were further categorized as either ‘non-severe’ or ‘severe’. Severe pulmonary infections were defined as those in which the patient required intensive care unit admission for treatment of pulmonary disease. Non-severe pulmonary infections included all other pulmonary infections.

We defined immunocompromise as the presence of any of the following documented conditions or medications in a patient at initial presentation for cryptococcal disease: active hematologic malignancy, recent neutropenia (defined as absolute neutrophil count <500 cells/µL in the 30 days before cryptococcal diagnosis), history of allogeneic or autologous stem cell transplant, solid organ transplant, cytotoxic chemotherapy, HIV infection, autoimmune disease, oral or parenteral steroid use at a dose >5 mg/day during the past year, or current use of other drugs that suppress the immune system (i.e. methotrexate, tumor necrosis factor-α inhibitor). We defined major medical comorbidities as existence of pulmonary, cardiac, liver or renal disease, documented diabetes, or immunocompromise as defined above.

RIT was defined, based on 2010 and 2000 IDSA guidelines, as the administration of AMB/5FC for CNS infections, severe pulmonary infections, and bloodstream infections, and administration of an azole drug (fluconazole, itraconazole, or posaconazole) for patients with non-severe pulmonary infections [34], [35]. Alternative initial treatment (AIT) included any other initial antifungal treatment for the respective infections. Recognizing that clinical information obtained during the days following a patient’s diagnosis with cryptococcosis might impact clinical decision-making, we assessed whether treatment was RIT or AIT at four days after a diagnosis of *C.gattii* was made. For example, for patients with severe pulmonary disease for whom RIT included AMB/5FC, an alternate treatment during days 1–4 after diagnosis did not result in an AIT classification if the patient was switched to AMB/5FC by day five. However, continued AIT past the four-day mark would result in a patient being designated as receiving AIT.

### Data Analysis

Comparisons of proportions were evaluated with the χ^2^ test; the Fisher’s exact test was used when one or more cell counts were <5. Comparison of medians was done with the Wilcoxon-rank-sum test. All analysis was done in SAS version 9.3 (SAS Institute Inc., Cary, NC).

## Results

### Demographics

We identified 74 patients with invasive *C. gattii* infections: 19 (26%) in Washington and 55 (74%) in Oregon. Four patients died before diagnosis of *C. gattii* infection (3, 7, 7, and 12 days before diagnosis); two had bloodstream infections and two had pulmonary infections (both severe).

Seventy (95%) patients survived to diagnosis and were included in further analysis. Median time from symptom onset to diagnosis was 34 (range: 3–351) days. Median patient age was 54 (range: 15–96) years; 36 (51%) were female. Sixty-five (93%) patient isolates were identified as outbreak-strain VGII subtypes, with 43 (61%) VGIIa, 17 (24%) VGIIc, and five (7%) VGIIb; of the remaining isolates, four (6%) were molecular type VGI and one (1%) was VGIII. Fifty-seven (81%) patients were hospitalized at the time of cryptococcal diagnosis. Of the 69 patients with immune status documented, 35 (51%) were immunocompromised at presentation. The most common immunocompromising conditions were systemic steroid use (24 patients; 69%) and autoimmune disease (13 patients; 37%). Among all 70 patients who survived to diagnosis, 3 (4%) patients had documented HIV infection; 36 (51%) additional patients had documented testing for HIV infection at the time of diagnosis of *C. gattii* infection and were found to be negative. Non-immunocompromising comorbid conditions were also common: 29 (41%) patients had cardiovascular disease, 16 (23%) had diabetes, and 14 (20%) had underlying respiratory disease. Nine (13%) patients were otherwise healthy (no immunocompromise and no comorbid conditions). Thirteen (19%) patients died within three months of diagnosis (Table 1).

**Table 1:** Characteristics of patients with invasive *Cryptococcus gattii* infection in United States Pacific Northwest who survived to diagnosis (N = 70).

Characteristic	Sub-category	N (%)
Female		36 (51)
VGII molecular type isolates^a^		65 (93)
Median age (range) in years		54 (15–96)
Immunocompromise^b^		35 (51)
	Systemic steroid use^c^	24 (69)
	Autoimmune disease^c^	13 (37)
	HIV^c^	3 (4)
Hospitalized at cryptococcal diagnosis		57 (81)
Medical co-morbidity^c^	Cardiovascular disease	29 (41)
	Diabetes	16 (23)
	Respiratory disease	14 (20)
Otherwise healthy (no immunocompromise or comorbid conditions)		9 (13)
Site of infection	Pulmonary	33 (47)
	CNS	30 (43)
	Bloodstream	7 (10)
Severity of pulmonary infection	Severe	9 (27)
	Non-severe	24 (73)
Median time from symptom onset to diagnosis in days		34 (3–351)
Died within 3 months of diagnosis		13 (19)

a VGII molecular type isolates include isolates from the three outbreak genotypes, VGIIa, VGIIb, and VGIIc.

b n = 69.

c Categories not mutually exclusive.

### Sites and Severity of Infection

For the purposes of this analysis, 33 (47%) of the 70 patients surviving to diagnosis were categorized as having pulmonary infections, 30 (43%) were categorized as having CNS infections, and seven (10%) were categorized as having bloodstream infections. Of the 33 patients with pulmonary infections, 24 (73%) infections were non-severe and nine (27%) were severe (Table 1). Table 2 shows all documented sites of *C. gattii* infection for all patients and the infection-type categorization used during this analysis.

**Table 2:** All documented sites of *Cryptococcus gattii* infection and categorization for analysis*.

Body sites found to have *Cryptococcus gattii*infection during clinical workup	Categorization of infectiontype for analysis	Number of patients
Lungs	Pulmonary	33
Blood	Bloodstream	2
Blood/Central Nervous System	Bloodstream	4
Blood/Central Nervous System/Lungs	Bloodstream	1
Central Nervous System/Lungs	CNS	5
Central Nervous System	CNS	25

*Total patients in analysis with pulmonary infection, 33; with bloodstream infection, 7; with CNS infection, 30.

While most immunocompromised patients had pulmonary infections (20/35, 57%), most immunocompetent patients had CNS infections (18/34, 53%). Time from symptom onset to diagnosis of *C. gattii* infection was significantly longer among patients with pulmonary infections (50 days) than those with either CNS (24 days) (p = 0.005) or bloodstream infections (27 days) (p = 0.02). There were no differences in immune status between patients with bloodstream infections and either pulmonary or CNS infections.

### Treatment and Outcomes

Of the 70 patients surviving to diagnosis, 50 (71%) received RIT and 20 (29%) received AIT. Three (43%) patients with bloodstream infections received AIT, compared with 12 (36%) patients with pulmonary infections and five (17%) patients with CNS infections (Table 3). More patients with pulmonary (36%) than CNS (17%) infections received AIT, though this difference was borderline significant (p = 0.07) (Table 3). Patients with bloodstream infections were not significantly more likely than those with either pulmonary (p = 1.0) or CNS (p = 0.16) infections to receive AIT; however, the small number of patients with bloodstream infections likely limited our ability to compare these groups.

**Table 3:** Three-month mortality among patients by site of infection and by initial therapy received*.

Sites of infection	n	Receivedrecommendedinitial treatment	3-month mortality amongthose receivingrecommended initial therapy	Receivedalternativeinitial therapy	3-month mortality amongthose receiving alternativeinitial therapy
All	70	50 (71%)	7 (14%)	20 (29%)	6 (30%)
**By site of infection**					
Pulmonary	33	21 (64%)	2 (10%)	12 (36%)	6 (30%)
CNS	30	25 (83%)	3 (12%)	5 (17%)	0 (0%)
Bloodstream	7	4 (57%)	2 (40%)	3 (43%)	1 (50%)
**By severity of pulmonary infection**					
Severe pulmonary	9	1 (11%)	0 (0%)	8 (89%)	4 (17%)
Non-severe	24	20 (83%)	2 (2%)	4 (17%)	2 (50%)

*Mortality measured from date of diagnosis; 4 patients died prior to diagnosis and receipt of antifungal therapy and are not included in this table.

Among patients with pulmonary infections, those with severe infections were more likely to receive AIT than those with non-severe infections (89% vs. 17%, p<0.001) (Table 3). Of the eight patients with severe pulmonary infections receiving AIT, seven (88%) received an azole only and one (12%) received AMB monotherapy (no 5FC given) (Table 4). Of the four patients with non-severe pulmonary infections who received AIT, one (25%) received AMB monotherapy, one (25%) received caspofungin and voriconazole, and two (50%) received no treatment (Table 4). All five patients with CNS infections and all three patients with bloodstream infections who received AIT received AMB monotherapy (no 5FC given) (Table 4).

**Table 4:** Initial therapy received by *Cryptococcus gattii* patients.

Site of infection	n	Recommended initial therapy (n)	Alternative initial therapy (n)
Severe pulmonary	9	Amphotericin B/5-flucytosine (1)	Azole only (7); Amphotericin B only (1)
Non-severe pulmonary	24	Fluconazole (20)	No treatment (2); Amphotericin B only (1);caspofungin/voriconazole (1)
CNS	30	Amphotericin B/5-flucytosine (25)	Amphotericin B only (5)
Bloodstream	7	Amphotericin B/5-flucytosine (4)	Amphotericin B only (3)

Thirteen (19%) of the 70 patients surviving to diagnosis died within three months. Three-month mortality was highest for patients with bloodstream infections (3/7; 43%), next-highest for patients with pulmonary infections (7/33; 21%), and lowest for patients with CNS infections (3/30; 10%). Overall, three-month mortality was non-significantly higher among patients receiving AIT compared with those receiving RIT (30% vs. 14%, p = 0.12), driven primarily by a trend in increased mortality among patients with pulmonary infections receiving AIT versus RIT (42% vs. 10%, p = 0.07) (Table 3). The increased three-month mortality among patients with pulmonary disease receiving AIT versus RIT was observed both for patients with severe (38% vs. 0%, p = 1.0) and non-severe pulmonary disease (50% vs. 10%, p = 0.12) (Table 3). RIT was not associated with increased mortality among patients with CNS or bloodstream infections (p = 1.0 for both). No association was found between three-month mortality and immunocompromising conditions; nine (26%) immunocompromised patients died within three months of their diagnosis, compared with four (12%) immunocompetent patients (p = 0.22). Similarly, no association was found between three-month mortality and presence of any pre-existing major medical comorbidity; ten (16%) patients with pre-existing comorbidities died within three months of their diagnosis, compared with three (33%) without any pre-existing condition (p = 0.35). No association was found between three-month mortality and time to diagnosis (p = 0.68).

## Discussion

We describe the initial antifungal treatments utilized in United States Pacific Northwest *C. gattii* infections and subsequent patient outcomes. Patients in this analysis, as previously described [16], [33], were frequently immunocompromised or had serious comorbid conditions and most commonly presented with pulmonary disease. The overall case-fatality rate for this cohort was high (19%). We found that while a substantial minority of patients did not receive IDSA guideline-recommended initial therapy, the receipt of alternative initial treatments was not equally distributed across all *C. gattii* infections. Fewer patients with pulmonary infections compared with central nervous system infections received IDSA guideline-recommended initial therapy. Among patients with isolated pulmonary infections, fewer with severe pulmonary infections received recommended initial therapy compared with those persons with non-severe infections. Among the patients who received alternate initial treatment, most were ‘under-treated’, either through failure to receive 5-flucytosine with amphotericin B (for patients with CNS, bloodstream and severe pulmonary infections), or failure to receive any treatment (for patients with non-severe pulmonary infections). Receipt of an alternative initial therapy was associated with a non-significant trend towards increased mortality (approximately 30% excess mortality) in the three months after diagnosis, particularly among patients with pulmonary infections.

There are a number of reasons why IDSA-recommended initial therapy might not have been used with patients in this cohort. While infectious disease clinicians are likely to be aware of the IDSA guidelines for cryptococcal disease, many patients are initially treated by clinicians without formal infectious disease training who may not be aware of the IDSA guidelines. Specifically, they may not be aware that severe pulmonary cryptococcosis should be treated in the same way as central nervous system cryptococcosis, leading to under-treatment of patients with severe pulmonary infections. Additionally, as *C. gattii* infections in the United States Pacific Northwest appear to be clinically different from *C. gattii* infections in other areas of the world, some clinicians who are aware of the IDSA guidelines in Oregon and Washington State may initially deviate from IDSA-recommended therapy due to concerns about the generalizability of the guidelines to their patients with *C. gattii* infection. Finally, clinicians may not have used guideline-recommended initial therapy due to matters beyond their control, such as patient contraindications to medications, insurance restrictions, or drug shortages. While we were unable to evaluate why clinicians chose, in a minority of patients, to pursue alternative treatments, our data suggests that there might be some benefit in adhering to IDSA guideline-recommended initial treatment in United States Pacific Northwest *C. gattii* patients, particularly those with pulmonary disease. Further research into the reasons for use of alternative initial treatment regimens is needed.

Pulmonary cryptococcosis presents a number of clinical challenges in diagnosis and treatment. Unlike cryptococcal meningitis, a common HIV-related opportunistic infection, pulmonary cryptococcosis is much less-commonly-recognized clinical entity, even among HIV-infected persons [36]. Diagnostic delays for patients with pulmonary cryptococcosis, as seen in this cohort, have been documented previously [37]–[40]. In terms of treatment, while IDSA guidelines do specify use of antifungal treatment for pulmonary cryptococcal infections – even mild disease - and the use of amphotericin B and 5-flucytosine in ‘severe’ pulmonary disease [34], [35], the quality of the evidence for both recommendations is limited and based on ‘…opinions of respected authorities…clinical experience, descriptive studies, or reports of expert committees’ [34]. Unlike for cryptococcal meningitis [41]–[44], no randomized controlled trials evaluating best treatments for pulmonary cryptococcosis have been published, and divergent opinions exist in the literature on the utility and optimal type of antifungal treatment for these patients. Some clinicians have suggested that asymptomatic or minimally symptomatic pulmonary cryptococcosis in immunocompetent persons requires no antifungal treatment at all [45], [46], while others have recommended azole drugs or amphotericin B in all cases [47], [48]. In Australia, where guidelines recommend amphotericin B and 5-flucytosine for all but mild/asymptomatic pulmonary cryptococcosis, Chen *et al* recently published outcomes data on ten patients with isolated pulmonary *C. gattii* infection. The majority of patients were treated with amphotericin B and 5-flucytosine and only one death was reported [49], raising the question of whether a more aggressive approach might be warranted among patients with pulmonary *C. gattii* infections. Larger-scale evaluations of patients with pulmonary cryptococcal infections, including patients with *C. gattii* infections from both previously-recognized endemic areas and the United States Pacific Northwest, are needed to identify the most appropriate treatment and improve outcomes.

We chose to evaluate initial antifungal treatment, and not treatment later in the course of disease, for several reasons. First, initial treatment, termed induction therapy, for cryptococcal disease has been shown to have a strong impact on mortality. Studies of HIV-infected patients during the early years of the HIV epidemic demonstrated that induction therapy for cryptococcal meningitis with fluconazole resulted in worse outcomes than induction with amphotericin B and 5-flucytosine [46]. Similarly, treatment with amphotericin B alone (i.e., without 5-flucytosine) has been shown to be inferior to combination amphotericin B and 5-flucytosine therapy for induction [44], [45]. In addition, we believed that selection of the initial antifungal drug is less likely than subsequent treatment choices to be influenced by outside factors (e.g., patient contraindications, adverse reactions) and therefore more indicative of physician preference and knowledge. However, it is possible that consolidation and maintenance drug choices as well as duration of therapy also may influence patient outcomes. Unfortunately, we were not able to evaluate that relationship in this study.

This analysis included several limitations. First, this patient group included those with *C. gattii* infections that were severe enough to required hospitalization; thus, our findings regarding treatment and outcomes are likely not applicable to mild, self-limited pulmonary *C. gattii* infections. However, few of these mild infections have been identified in this cohort and it is unclear how frequently they occur. Second, due to the retrospective nature of this study, not all patients received identical diagnostic testing (e.g., lumbar puncture, chest imaging, bronchoalveolar lavage); this may have led to incomplete ascertainment of all sites of infection. Third, these results are specific to patients with *C. gattii* infection in the United States Pacific Northwest, and may not be generalizable to patients with *C. gattii* infection in other areas. Finally, the number of patients in our evaluation was small, particularly in subgroup analyses. More data, ideally from prospective studies or clinical trials, is needed to understand the relationship, if any, between site of infection, initial antifungal treatment, and outcomes in this population.

This is the first evaluation of the effect of initial antifungal treatment on patient outcomes in the North American outbreak of *C. gattii*. We show that a substantial minority of patients are not getting the current guideline-recommended initial antifungal therapy, which may be associated with improved outcomes. Timely diagnosis and appropriate treatment for patients with *C. gattii* infection will continue to be a clinical question as the emergence of *C. gattii* in Oregon, Washington State, British Columbia, and elsewhere continues. *C. gattii* infections in persons with and without recent travel history to the United States Pacific Northwest or British Columbia are increasingly being reported throughout the United States [14], [29], [33], [50], [51]; this increased visibility will raise more questions about the best treatment for patients with *C. gattii*. As our identification of *C. gattii* infections improves and diagnoses increase, as they are likely to do, careful collection of treatment-related data from patients with these infections will be vital to improving outcomes.
